# Fast Traffic Sign Recognition with a Rotation Invariant Binary Pattern Based Feature

**DOI:** 10.3390/s150102161

**Published:** 2015-01-19

**Authors:** Shouyi Yin, Peng Ouyang, Leibo Liu, Yike Guo, Shaojun Wei

**Affiliations:** 1 Institute of Microelectronics, Tsinghua University, Beijing 100084, China; E-Mails: oyangpeng12@163.com (P.O.); liulb@tsinghua.edu.cn (L.L.); wsj@tsinghua.edu.cn (S.W.); 2 Department of Computing, Imperial College, London SW7 2AZ, UK; E-Mail: y.guo@imperial.ac.uk

**Keywords:** traffic sign recognition, binary pattern, SIFT, artificial neutral network

## Abstract

Robust and fast traffic sign recognition is very important but difficult for safe driving assistance systems. This study addresses fast and robust traffic sign recognition to enhance driving safety. The proposed method includes three stages. First, a typical Hough transformation is adopted to implement coarse-grained location of the candidate regions of traffic signs. Second, a RIBP (Rotation Invariant Binary Pattern) based feature in the affine and Gaussian space is proposed to reduce the time of traffic sign detection and achieve robust traffic sign detection in terms of scale, rotation, and illumination. Third, the techniques of ANN (Artificial Neutral Network) based feature dimension reduction and classification are designed to reduce the traffic sign recognition time. Compared with the current work, the experimental results in the public datasets show that this work achieves robustness in traffic sign recognition with comparable recognition accuracy and faster processing speed, including training speed and recognition speed.

## 1. Introduction

**Figure 1.**
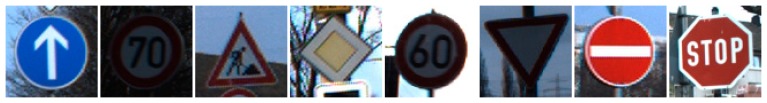
Different kinds of traffic signs from GTSRB data set.

With the development of the intelligent car [1–3], safe driving assistance systems are becoming more and more important. In the safe driving assistance system, traffic sign recognition is a key technology, and has been widely used [4,5]. The accuracy and short processing time are extremely important for traffic sign recognition. However, in practical driving conditions, the diverse situations of traffic signs including the rotation, viewpoint, scale and illumination are complex and undesirable. As shown in Figure 1, some traffic signs in variable conditions are illustrated. Achieving robust traffic sign recognition with short processing times is a very challenging endeavor. Traffic sign recognition includes traffic sign detection and traffic sign classification. In order to achieve fast and robust traffic sign detection, designing a computing efficient and highly discriminative feature is essential. Meanwhile, in order to achieve fast and robust traffic sign classification, establishing a classification process that can reduce the amount of features and sustain classification accuracy is also very important.

Recently, many works have focused on traffic sign detection and recognition [6]. Some works achieve traffic sign detection and recognition by designing lots of pre-processing methods [7–10]. These works can achieve fast processing speed, but lack the generalization capability or robustness to recognize traffic signs in different and complex conditions. Other works adopt the classifiers to implement a training process for the classifiers to enhance the recognition of traffic signs based on large amounts of training samples [11–15]. Especially, the deep learning method is adopted to distinguish features from the pixels [15]. Though these classifications based methods achieve high classification accuracy, the learning and classification time is large, and hence should be further optimized for real safety assistance driving systems.

Considering the processing time and classification accuracy as a whole, the specifically designed features for traffic sign detection, together with computing efficient classification, have been researched [16,17]. When designing the specific features, the conditions existing in different driving scenes including the rotation, illumination and scale should be taken into account to achieve robust detection. Meanwhile, the computing time of these features should be reduced. However, these works lack such kinds of design considerations. The typical ASIFT (Affine Scale Invariant Feature Transform) feature, SIFT (Scale Invariant Feature Transform) feature and SURF (Speed Up Robust Feature) feature exhibit highly discriminative performance [18–20], and have much potential to achieve robust traffic sign detection. Furthermore, among the ASIFT, SIFT and SURF, ASIFT exhibits the highest discriminative performance. However, these algorithms—ASIFT, SIFT and SURF—are all time consuming.

In this work, in order to achieve robust and fast traffic sign detection, a rotation invariant binary pattern based feature in the affine space and Gaussian apace is proposed. This specific feature leverages the techniques from ASFIT to achieve robustness in scale, rotation and illumination, meanwhile improving the computation efficiency. Further, in order to reduce the classification time, the methods of ANN (Artificial Neural Network) based feature dimension reduction and classification are adopted, which turn the complex clustering and matching processing into a small amount of parameter computation. Experimental results show that this work attains robust traffic sign recognition in comparison to the state-of-the-art methods, and achieves a faster processing time, including training time and classification time.

The rest of the paper is organized as follows. Section 2 introduces the current research on traffic sign detection and recognition. Section 3 introduces the proposed rotation invariant binary pattern based feature and the entire approach for traffic sign recognition. The evaluation of results is presented in Section 4. The last section concludes the paper and outlines future directions.

## 2. Related Work

Current works on the traffic sign detection and recognition can be divided into three categories. First, pre-processing methods are researched to locate and recognize the traffic signs. Second, pre-processing methods combining with classification are adopted to achieve robust traffic signs recognition. Third, specific design features combing with the classifiers are used to achieve the robust and computing efficient recognition. These three categories are introduced below.

First, many methods achieve the robust traffic sign detection and recognition by designing robust pre-processing methods [7–10]. Siogkas [7] proposes a complete automatic system for traffic sign detection and recognition by processing the video frames in L*a*b color space. Therein, L denotes the luminance, and a and b denote the color. Coronado [8] develops an intelligent system to achieve automatic traffic sign recognition in terms of dealing with the difficulties that arise from changes in lighting conditions and various obstacles. Hu [9] achieves the traffic sign detection based on the visual attention model. Larsson [10] adopts the Fourier descriptor to achieve traffic sign recognition. These methods require lots of pre-processing of the traffic images, and their generalization capability and robustness are limited.

Second, based on the methods of machine learning, many classifiers including deep learning have been used to achieve robust recognition [11–15]. Kuo [11] adopts the two stage classification strategy to achieve traffic sign detection and recognition. Ciresan [12] and Sermanet [13] adopt the convolution neural network to learn the discriminative features from the pixels. These works achieve high classification accuracy, but the time complexity of training and classification is extremely high. Lu [14] proposes a sparse representation based graph embedded method to learn of a subspace by means of the structures of traffic signs, and then adopts the sparse representation classifier to implement classification. Jin [15] proposes a hinge loss stochastic gradient method to train the convolution neural network based deep learning, which achieves the high recognition accuracy. However, these methods' learning features use the pixels or structures of the traffic signs based on large amounts of traffic samples bringing high computation complexity, while the training and the classification times are very high, which cannot sufficiently meet the requirements of intelligent driving systems.

Third, the specifically designed features achieve more advantages in reducing the computation complexity. Greenhalgh [16] and Zaklouta [17] consider the computing time cost and propose the method for real time traffic sign recognition. Greenhalgh [16] uses the maximally stable extremal regions (MSER) [21], and then implements the SVM (Support Vector Machine) based classification. Zaklouta [17] uses different sized HOG (Histograms of Oriented Gradients) features, and adopts random forest based classification to achieve high detection accuracy. Tang [22] proposes an efficient method of traffic sign recognition using complementary features to reduce the computation complexity of traffic sign detection, and then use the SVM to implement the traffic sign classification. The complementary features used in Tang [22] include HOG [23] and LBP (Local Binary Pattern) [24]. However, HOG and LBP features used in these methods cannot simultaneously tackle the situations of rotation, viewpoint, scale and illumination well. The typical ASIFT feature, as the most discriminative feature in the current feature works, can tackle rotation, viewpoint, scale and illumination very well [20], and hence has the most potential for traffic sign detection.

According to the ASIFT work [20], the ASIFT algorithm simulates the original image by rotation and tilt transformation, and then implements the SIFT algorithm on these simulated images. The SIFT (Scale Invariant Transform) feature generation mainly includes Gaussian pyramid, extrema localization and feature generation. Because of the Gaussian pyramid and local rotation invariant processing by accumulating the orientations and magnitude, the SIFT algorithm exhibits strong advantages on the rotation and scale invariant. The ASIFT algorithm can perform equally to SIFT when implemented on the different affine simulations of the same image. Hence, it achieves high robustness in the affine space. However, the SIFT feature is based on the local intensity of the pixels; though the normalization for the feature vector achieves the illumination invariant, the performance is decreased when used in complex situations with large changes in illumination. Besides, since more images need to be processed by the SIFT algorithm and the SIFT algorithm itself is characterized by high computation complexity, the computation complexity for the ASIFT is high, which inhibits ASIFT from being directly implemented in traffic sign detection.

In this work, in order to achieve high robustness in traffic sign detection, the techniques of affine simulation, main orientation computation and extrema localization in the ASIFT are reused, and a new feature that is computing efficient is proposed based on ASIFT for robust traffic sign detection. Meanwhile, the efficient ANN based classification is designed for traffic sign recognition to further enhances the robustness.

## 3. Proposed Method

This work uses the typical Hough transformation to locate the candidate regions of the traffic signs; and then implements two optimizations in the feature detection and classification, respectively. In this section, the first optimization called RIBP (Rotation invariant binary pattern) based feature algorithm is explained in Subsection 3.1. Then, the second optimization called ANN (Artificial Neural Network) based feature dimension reduction and classification is introduced in Subsections 3.2 and 3.3, respectively. The processing flow for the traffic sign recognition is illustrated in Subsection 3.4.

### 3.1. Rotation Invariant Binary Patter Based Feature

Since the histogram of binary patterns is illumination responsive and exhibits high discrimination [25,26], this work proposes scale and rotation invariant circular binary pattern based histograms to generate the feature vector. The main idea includes two aspects. First, we build the minimal rotation invariant binary pattern (RIBP) image based on the original image block to enhance the illumination robustness; second, we construct the rotation invariant binary pattern based circular histogram to enhance the rotation invariant. All the analysis in this section is based on two design considerations. First, after the affine simulation and Gaussian pyramid, two pictures are placed approximately in the same scale and position; second, local filtering filters the extreme noises and ensures the stable change of the illumination.

Define the image intensity as *I*(*x*, *y*) in the location (*x*, *y*), where *x* ∈ [0, *w* −1] and *y* ∈ [0, *h* −1]. and *h* denote the image width and image height of image, respectively. The illumination change in affine space is modeled in Equation (1).

(1) $$I^{`}(x,y)=\alpha (\sum_{i=1}^{K}w_{i}I(x+d_{x}^{i},y+d_{y}^{i}))+\beta$$

where *w_i_*, *α* and *β* denote the weighting factor, scaling factor and shifting factor for the intensity, respectively; $d_{x}^{i}$, $d_{y}^{i}$ denote the location shift in *x* orientation and *y* orientation; *k* denotes the number of the pixels in the original image to attend the transformation for the pixel *I′*(*x*, *y*) in the affine transformed image.

In this work, the Gaussian pyramid technique and affine simulation technique are adopted to ensure the affine and scale invariant. After affine simulation, the image pair that is approximately similar is achieved. Hence, the original expression in Equation (1) is rewritten in Equation (2).

(2) $$I^{`}(x,y)\approx \alpha I(x+d_{x},y+d_{y})+\beta$$

Since the binary pattern mode can achieve robustness in illumination [25,26], given the pixels *I*(*x*, *y*) and, *I`*(*x*+1, *y*+1), assume *I′*(*x, y*) > *I`*(*x*+1, *y*+1), we can achieve the expression in Equation (3) according to Equation (2):

(3) $$\alpha I(x+d_{x},y+d_{y})+\beta >\alpha I(x+1+d_{x},y+1+d_{y})+\beta$$

Equation (3) can be simplified as Equation (4):

(4) $$I(x+d_{x},y+d_{y})>I(x+1+d_{x},y+1+d_{y})$$

Then, construct the binary pattern mode to denote the relationship of intensity value, for which the relationship shown in Equation (5) is achieved by means of binary pattern mode transformation *ψ*(*para*1, *para*2) mentioned in [25,26].

(5) $$\psi (I^{`}(x,y),I^{`}(x+1,y+1))=\psi (I(x+d_{x},y+d_{y}),I^{`}(x+1+d_{x},y+1+d_{y}))$$

In Equation (5), *ψ*(*para*1, *para*2) denotes 1 when *para*1 > *para*2, and denotes 0 when *para*1 ≤ *para*2. *para*1 and *para*2 denote the parameters in the function *ψ*. According to this transformation, the illumination disturbance of the pixels can be well handled.

In Equation (2), the relationship is of approximate equivalence. In order to reduce the approximating error, we first use the method of intensity accumulation which is shown in Equation (6).

(6) $$\sum_{-1\leq i,j\leq 1}I^{`}(x+i,y+j)\approx \sum_{-1\leq i,j\leq 1}\alpha I(x+i+d_{x},y+j+d_{y})+\beta$$

Then, construct the binary pattern image based on the original image block to enhance the illumination robustness according to Equation (7).

(7) $$\begin{array}{l} \psi (\underset{-1\leq i,j\leq 1}{\text{∑}}I^{`}(x+i,y+j),\underset{-1\leq i,j\leq 1}{\text{∑}}I^{`}(x+i+b_{\text{width}},y+j+b_{\text{width}}))=\\ \psi (\underset{-1\leq i,j\leq 1}{\text{∑}}I^{`}(x+d_{x}+i,y+d_{y}+j),\underset{-1\leq i,j\leq 1}{\text{∑}}I^{`}(x+d_{x}+i+b_{\text{width}},y+d_{y}+j+b_{\text{width}})) \end{array}$$

Then, according to Equation (8), we rotate the binary pattern around the central pixel to the minimal value to achieve a stable descriptor for the center pixel, and meanwhile the rotation invariant is improved for the center pixel.

(8) $${\left(\psi_{1}(•),\psi_{2}(•),\ldots \ldots \psi_{8}(•))\overset{\text{shift}}{\to }(\psi_{z}(•),\ldots \ldots \psi_{8}(•),\ldots \right)}_{\text{min}}$$

**Figure 2.**
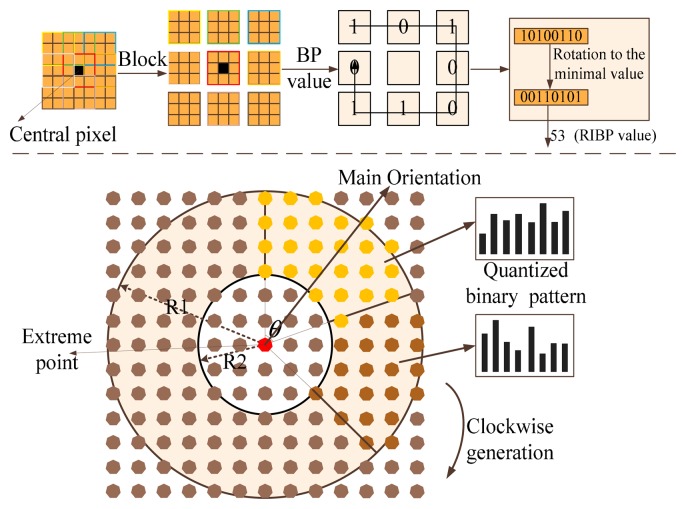
Illustration of the rotation invariant binary pattern based feature computing to achieve fast and robust traffic sign detection.

The whole process for the Equations (6)–(8) is shown in the top part of Figure 2, and based on this transformation, the final RIBP (Rotation invariant binary pattern) based image is generated. Since this process mainly contains the operations of addition and comparison to achieve a binary result, the computing speed can be improved.

Further, as shown in the bottom part of Figure 2, the rotation invariant BP based circular histogram is illustrated. Define *R*(*R*1, *R*2) as the regions between *R*1 and *R*2 in the circular regions, where *R*1 denotes the outer radius and *R*2 denotes the inner radius. Define θ as the angle occupied by each ring section. Define *Bin* as the number of the statistical classifications in the histogram. In this work, the local main orientation computation and extreme localization are computed based on the techniques in the SIFT algorithm. When obtaining the main orientation and extreme location, this method constructs the ring section according to the main orientation and the binary pattern value to generate the histogram in region of *R*(*R*1, *R*2). The center of the initial ring section is located in the main orientation, and several ring sections construct the joint circular histograms. The number of ring sections is *RN* = [(2*PI)/*θ*].

Based on the analysis mentioned above, we can compute the feature vector: *Fea* = {*F*_0_, *F*_1_,…*F_RN_*_−1_}, where *F_j_* = {*c*_1_, *c*_2_,…*c_bin_*}, and *F_j_* is the statistics of the rotation invariant binary patterns, and *c*_1_, *c*_2_….*c_bin_* denote the statistics of RIBP in different histogram region. Given the main orientations *O*_1_ and *O*_2_, the feature vectors for the correspondences in the two images are computed as shown in Equations (9) and (10). Note that these two main orientations are computed for the same correspondence in the original image and the affine transformed image, respectively.

(9) $$\text{Fea}1=\{F_{([O_{1}\times RN/2\times PI]+0)\%RN},\ldots F_{([O_{1}\times RN/2\times PI]+RN-1)\%RN}\}$$

(10) $$\text{Fea}2=\{F_{([O_{2}\times RN/2\times PI]+0)\%RN},\ldots F_{([O_{2}\times RN/2\times PI]+RN-1)\%RN}\}$$

Though the main orientations are different for the feature pair, the rotation invariant histograms for the RIBP in the circle region achieves highly invariant rotation. The relationship of these two features is |*Fea*1 − *Fea*2|≤ *τ*, where *τ* is a small threshold value for comparison. This optimization goal is to minimize *τ* to achieve the highest robustness. The optimization is shown in Equation (11).

(11) $$\begin{array}{c} \mathrm{Min}(\tau )\\ s.t.|\text{Fea}1-\text{Fea}2|\leq \tau \\ \phi (R1,R2,\text{Bin},\theta )<T_{\max} \end{array}$$

|*Fea*1 − *Fea*2|≤ *τ* shows the constraint for robust detection. The parameters: *R*1, *R*2, *Bin* and *θ* affect the discrimination performance and computing time, *ϕ*(*R*1, *R*2, *Bin*, *θ*) denotes the computing time when choosing the parameter: *R*1, *R*2, *Bin* and *θ*. *T_max_* denotes the allowed maximal computing time. In a real safe driving assistance system, *T_max_* should guarantee the real time processing. *ϕ*(*R*1, *R*2, *Bin*, *θ*) < *T_max_* shows the constraint for the time of feature computing.

**Figure 3.**
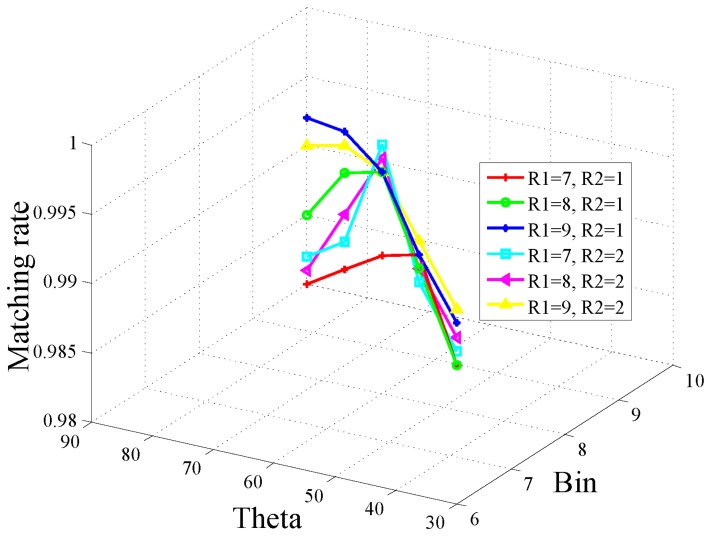
Matching rate.

**Figure 4.**
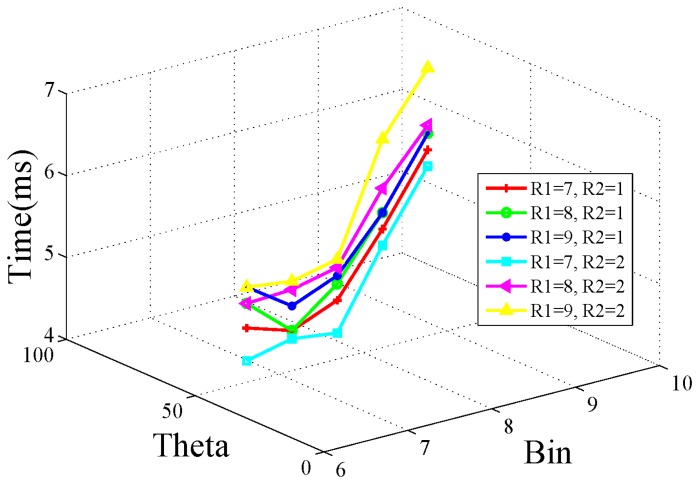
Processing time.

In this work, the experiments based on the public traffic sign dataset from [27] are implemented to search for the optimal parameters. The performance metric of matching rate (right matched points/total matched points) to verify the discrimination performance of this proposed feature is adopted. High matching rate denotes high discrimination performance, and also means the *τ* in Equation (11) tends to be minimal. The matching rate and processing time are averagely computed and these results are shown in Figures 3 and 4, respectively. In these experiments, six sets of (*R*1, *R*2) are tested; the *θ* (represented by Theta in the graph) ranges from 35 degrees–90 degrees with an interval of 15 degrees; the *Bin* value ranges 6–10 with interval of 1. The blue line (*R*1 = 7, *R*2 = 2) exhibits more advantages with high matching performance and low processing time. From Figure 4, we can see, with the blue line, the feature computing time is less than 5 ms when *θ* = *PI* = 3 and *Bin* = 8. In real safety driving assistance systems, if the feature computing time is less than 5 ms (which means *T_max_* = 5*ms*), it gives a high margin to the whole system for real time processing. Therefore, in this work, we set the parameters as, *R*1 = 7, *R*2 = 2, *θ* = *PI*/3 and *Bin* = 8.

### 3.2. Artificial Neural Network Based Feature Dimension Reduction

In this work, after the traffic sign detection using the proposed feature method, the feature vector with 64 fixed-point data is generated from the candidate regions. The number of the features detected from the candidate regions is uncertain, and it is determined by the image content in the candidate regions. Hence, the K-means clustering method that clusters these feature vectors into specific dimensions contribute to the reduction of time in traffic sign classification. However, the clustering process needs extra computation time. Different from the traditional way of directly clustering the features using the K-means method, this work uses the artificial neural network (ANN) based method to train the K-means to reduce the computation time of clustering. As shown in Table 1, these ANN based K-means methods are trained in the public traffic sign dataset from [27] and different topologies of the ANN affect the clustering accuracy. The ANN method includes three layers: input layer, hidden layer and output layer; and the ANN topology: a->b->c denotes the number of nodes in each layer. Setting the clustering of K-means as the baseline, the best topology for the ANN based K-means (topology: 16->16->1) achieves 600 times' improvement in the computation speed with only small 0.41% clustering accuracy loss. Note that, in these experiments, the features are unified as six clustering centers as they exhibit the high performance in classification accuracy and computation time. Furthermore, for these cluster centers, each center is represented by a 64 fixed-pointed vector.

### 3.3. Artificial Neural Network Based Classification for Traffic Sign Recognition

Based on these clustered feature vectors mentioned in Section 3.2, this work trains different ANNs for different types of traffic signs. This ANN based classification transforms the traditional classification method that uses feature vectors to implement the classification into this method with small parameter computation in the ANN; thus, the classification speed is greatly improved. We train the ANN topologies for the 43 classes in the public traffic sign dataset from [27] to achieve the best ANN topology and weight parameters. In the ANN training process, the overfitting problem has been considered. The strategies including the weighting decreasing in ANN training, iteration stopping criterion setting, and cross validations are adopted to avoid the overfitting problem. Besides, for fair comparisons with the reference works, we use the software development tool VC++ 2010 to code the algorithms, and implement them on the same hardware platform (Intel (R) Core(TM), Duo CPU, E7200@2.53GHz, 4 GB memory, and 32 bit operating system). The corresponding comparisons are shown in Tables 2 and 3.

As shown in Table 2, for different ANN topologies, different classification accuracies are achieved. Compared with the traditional classification methods including the SVM, random forest and full matching, the best topology for the ANN (16->16->1) in this work improves 20 times, 4 times and 4500 times on the processing speeds, respectively; meanwhile the classification accuracy of this work is high. In Table 2, different kernels (linear, Gaussian, sigmoid, *etc.*) for the SVM and different numbers of decision trees (from 1 to 9) in the random forest are tested, and the average results are computed. Additionally, the SVM and random forest based classifications have been used in work by Zaklouta [17] and Tang [22], respectively.

Further, we implement experiments changing the image size to evaluate the recognition accuracy and computing speed. The corresponding results are shown in Table 3, where the ANN topology (16->16->1) is selected since it exhibits higher performance among the three ANN topologies shown in Table 2.

As shown in Table 3, with the increase of the image size, the enhanced computing speed in comparison to other works becomes more significant. This work implements fast local feature detection and then uses the ANN based feature dimension reduction and classification. Our work demonstrates that computing speed can be very high when implementing the ANN based classification. Performance of the SVM based method based on global searching to implement the classification will be affected by image size. Though the full matching method achieves the highest classification accuracy, its computing speed is quite slow as the full matching brings high computing costs. Note that, in Table 3, the symbol CA denotes the classification accuracy, and the symbol CS denotes the computing speed. For Tables 2 and 3, the computing speed denotes the classification time, where the detection speed is excluded since we just evaluate the performance of different classifiers for our proposed features.

### 3.4. The Whole Method Illustration

**Figure 5.**
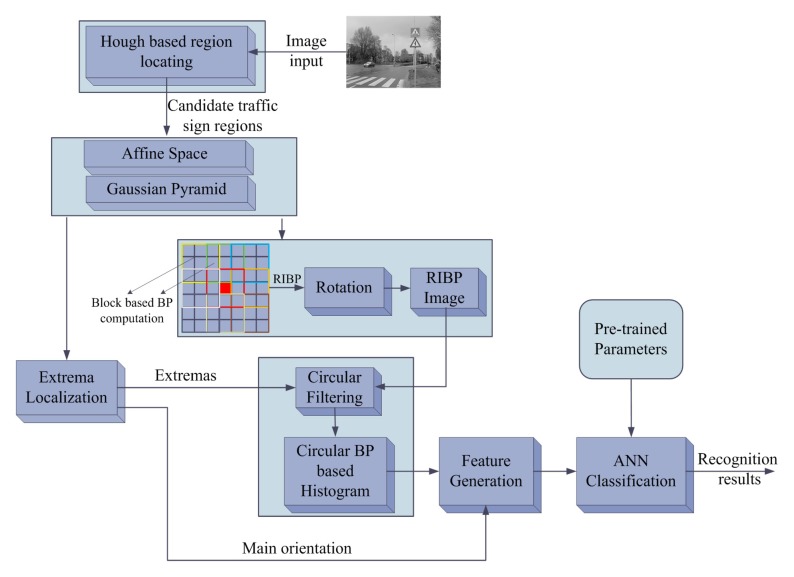
The whole computation flow of traffic sign recognition. The image embedded in the graph is from the GTSRB data set.

Based on the proposed feature and ANN based optimizations mentioned above, the whole method for the traffic sign recognition is shown in Figure 5. In Figure 5, the typical method of Hough transform (used in the OPENCV library) is adopted to locate the candidate regions for the traffic signs so that the candidate traffic sign regions will be focused to implement the feature detection. Testing on the public traffic sign dataset from [27] shows that locating the candidate regions in advance reduces the useless computations by 70% compared to that implementing feature detection overall images. The location of the candidate traffic sign regions is coarse-grained, and there are false candidate regions, which have the same shapes as the traffic signs. Hence, based on these candidate regions, this work implements fine-grained detection using the proposed features in this work.

As shown in Figure 5, the affine space and Gaussian pyramid technique are constructed to achieve scale robustness. The extreme localization technique is inherited from the SIFT algorithm [18]; it provides the main orientation and extrema coordinates. For the images in the Gaussian pyramid, this work generates the image block based binary pattern, and rotates the binary pattern sequence to the minimal position to generate the RIBP image. Then, the circular filtering for the circle region around the extreme point is implemented. Based on the extrema coordinates and main orientation, the rotation and scale invariant circular binary pattern based histogram is computed. Through the conjoined histogram, this proposed method quantizes them into the fixed-pointed data and generates the feature vectors. The final feature is a 64-dimension vector; each dimension is represented by the fixed-point data. These fix-point data based feature vectors contribute to the computation speed improvement. The generated feature vectors on one candidate region will be clustered using the ANN based K-means method, and then the ANN classifiers, which load the pre-trained ANN parameters of different types of traffic signs, will classify clustered data. The ANN based K-means feature dimension reduction and classification greatly improves the processing speed.

## 4. Performance Evaluation

In order to verify the discrimination performance and computation efficiency of the proposed feature for traffic sign detection, the experiments on the public available data set of traffic signs are implemented, and the current highly discriminative and computing efficient features are adopted for comparison. The performance in terms of the feature computation time and correctly matched points are analyzed. Further, based on this proposed feature, the experiments based on the publicly available data set to evaluate the proposed method of traffic sign recognition are also implemented. The state-of-the-art works for traffic sign recognition are adopted for comparison. The performance is analyzed in terms of the recognition accuracy and processing time. Besides, we further perform the testing in real conditions for our work. In this section, all the experiments are implemented on a PC (Intel (R) Core(TM), Duo CPU, E7200@2.53 GHz, 4 GB memory, and 32 bit operating system) platform and an embedded platform (TI DM6467: DSP (1 GHz) +ARM (500 MHz), 256 M 32 bit DDR2 memory).

### 4.1. Evaluation Methodology

**Figure 6.**
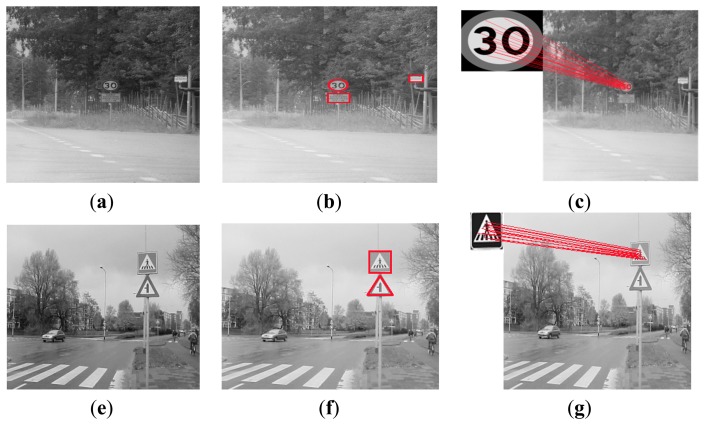
Parts of recognition results on GTSRB data set: (**a**) original image; (**b**) preprocessing to locate the candidate regions; (**c**) traffic sign recognition; (**e**) original image; (**f**) preprocessing to locate the candidate regions; (**g**) traffic sign recognition.

The public available dataset called German Traffic Sign Recognition Benchmark (GTSRB) [27] and Sweden traffic signs (STS) [11] are adopted for the performance evaluation. In the GTSRB dataset, there are 51,839 German traffic signs in 43 classes. These classes of traffic signs have been divided into six subsets [22]. These subsets include speed limit sign subset, unique sign subset, danger sign subset, mandatory sign subset, derestriction sign subset and other prohibitory sign subset. The size of these signs varies from 15 × 15 to 250 × 250. The images contain one traffic sign each, and each image contains a border of 10% around the actual traffic sign (at least 5 pixels) to allow for edge-based approaches. This dataset has the original size and locations of the regions of interests. Some graph results are shown in the Figure 6, where the two cases of traffic signs using our method to achieve the recognition are presented. The first case includes Figure 6a–c; the second case includes Figure 6e–g. Each case denotes the pre-processing and final recognition. The STS data set includes 20,000 images with 20% labeled and 3488 traffic signs. The label for each sign contains the sign type (pedestrian crossing, designated lane right, no standing or parking, priority road, give way, 50 kph, and 30 kph). The size of these signs ranges from 3 × 5 to 263 × 248, and the image size is 1280 × 960.

Further, the real traffic conditions are also considered for verification. In real traffic sign conditions, 25 video clips are recorded from the streets of Beijing by an on-board surveillance camera X6000. In these image datasets we collected, there are 200 Chinese traffic signs including the types of warring signs, direction signs, and forbidden signs. Meantime, the traffic signs' conditions in the night, daytime, raining and sunny day are considered in the training images and testing images to improve the classification performance. The evaluation results are shown in Section 4.5.

Besides, in this work, when implementing the ANN training process on these datasets, the strategies including weighting decreasing, iteration stopping criterion setting, and cross validations are used to avoid the ANN overfitting. For example, in the dataset of real traffic conditions, we use 5000 frame images with 1920 × 720 frame sizes to train the ANN classifier, and 2000 frame image to implement the testing.

In order to verify the discrimination performance and computing efficiency of the proposed feature, we select ASIFT as the baseline algorithm for the comparison since the ASIFT algorithm exhibits better discrimination performances than SIFT and SURF algorithms according to the affine SIFT work [20]. Besides, since the SURF is a highly computing efficient feature detector for SIFT and the core feature detector in the ASIFT is the SIFT algorithm, this work replaces the SIFT detector with the SURF detector for the ASIFT to construct the affine SURF (ASURF) algorithm to implement the performance comparisons for the feature processing time and correctly matched points. The results are computed based on averages. The comparisons with the ASIFT and ASURF for the correctly matched points and processing times including feature computation time and feature matching time are shown in Tables 4 and 5.

Besides, by means of combing the proposed ANN based techniques with the other features, the comparisons with the other features including the SIFT [18] and SURF [19], HOG [23], LBP [24], are implemented to further verify the whole method for traffic sign recognition. Note that the candidate regions for traffic signs and ANN classification are the same for these features, and the ANN topology is 16->16->1. The comparison results of the GTSRB data set and STS data set are shown in Tables 6 and 7, respectively.

**Figure 7.**
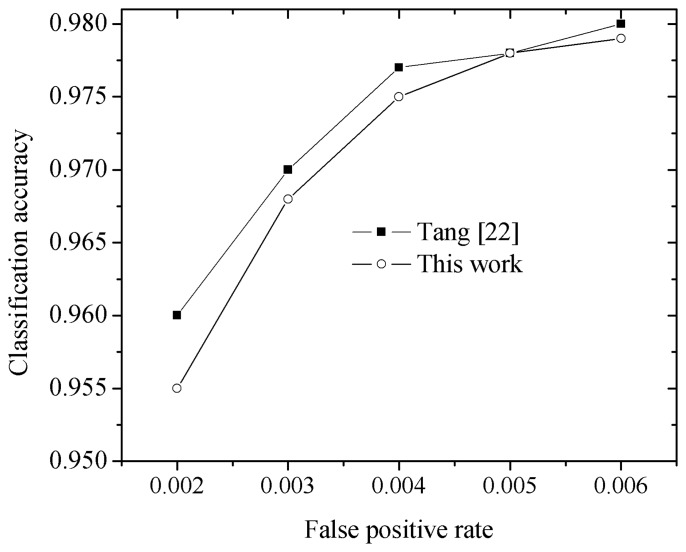
Testing in real conditions to obtain performance averages, and make a comparison with the work by Tang [22].

In order to verify the processing efficiency and recognition accuracy, state-of-the-art works including those by Larsson [10], Jin [15], Zaklouta [17] and Tang [22] are adopted. Larsson [10] uses the Fourier descriptor without classification to achieve traffic sign recognition. Jin [15] learns the features from the pixels of the traffic signs, whereby the time cost for the learning process is large. Zaklouta [17] and Tang [22] adopt a specifically designed feature for traffic sign recognition. Jin [15], Zaklouta [17] and Tang [22] implement their experiments for the GTSRB dataset to verify the performance; hence, as shown in Table 8, we implement our work for the GTSRB to achieve a fair comparison. Besides, Larsson [10] implemented the experiment for the STS data set. Hence, as shown in Table 9, we also make the comparison based on the STS data set. Considering all these works in terms of the classification accuracy and recognition time, Tang [22] performs better. Hence, we make a comparison with Tang [22] in real conditions, where the work from Tang [22] is re-implemented using the same hardware and software platform. The corresponding results are shown in Figure 7 and Table 10.

### 4.2. Feature Computing Performance Evaluation

In the proposed feature algorithm, the binary pattern based computations are represented by fixed-point data rather than the float-point data that was used in the other algorithms; hence, this proposed algorithm greatly reduces the computation amount. The normalization for the feature vector to improve the illumination robustness is removed in this work, which improves processing efficiency. The rotation invariant is based on the ring section based circular histograms, and reduces a lot of the computation of the pixel-level rotation in the SIFT and SURF. Besides, the length of the feature vector of the proposed feature is 64 dimensions, and each dimension is represented by a 7-bit data length; it reduces more than half of the representations in comparison to the ASIFT feature that is a 128-dimension vector, and achieves less bit representations than the SURF, though the SURF has the same vector dimensions as this work. In Table 4, we show the comparisons for processing times including the feature computation time and feature matching time for ASIFT, ASURF and this work. Note that, for verifying the feature computing performance in Table 4, the full matching method is used for feature matching, which is time consuming and makes the total processing time lengthy. In this work, the ANN based classification method is finally used to reduce the matching time.

As shown in Table 4, this work achieves, on average, 27.2 times' and 12.6 times' improvements in processing speed compared to the ASIFT and ASURF, respectively. Meanwhile, the corresponding results of correctly matched points in Table 5 show that this work experienced a small performance loss (averagely 6.9% less) compared to the ASIFT while exhibiting better performance (averagely 27% higher) than the ASURF. Note that more correctly matched points denote higher performance as more discriminative features are detected. Hence, as shown in Tables 4 and 5, this proposed feature greatly improves the processing time compared to the ASIFT and ASURF, while achieving comparable discrimination performance to the ASIFT and higher discrimination performance than the ASURF.

### 4.3. Performance Comparison with Typical Features Used in Traffic Sign Recognition

By combining ANN techniques with different features that have been used in the traffic sign detection in the previous works, this work further verifies the discrimination performance of the proposed feature when used in the entire process of traffic sign recognition. As shown in Table 6, for testing with the GTSRB dataset, this feature achieves the highest recognition accuracy, which reaches 98.62%. Meanwhile, this work reduces the training time by 1.9 times, 1.1 times, 2.3 times and 1.8 times, and improves the recognition speed by 3 times, 1 time, 199 times and 99 times, compared to the methods of HOG+ANN, LBP+ANN, SIFT+ANN and SURF+ANN, respectively.

As shown in Table 7, for testing with the STS data set, this feature achieves the highest recognition accuracy, which reaches 98.33%. Meanwhile, this work reduces the training time by 1.5 times, 1.1 times, 2.1 times and 1.1 times, and improves the recognition speed by 3.3 times, 1.5 times, 259 times and 103 times, compared to the methods of HOG+ANN, LBP+ANN, SIFT+ANN and SURF+ANN, respectively. Note that, in these experiments, the dimensions for the HOG and LBP are computed based on the image blocks. For the HOG feature, the image is divided into 7 × 7 blocks; each block has four cells and each cell use nine bins. Hence, the dimension is 36. For the LBP, the 59 dimension based features from the 6 × 6 blocks are extracted.

### 4.4. Performance Comparison with Other Traffic Sign Recognition Methods

According to the results for the GTSRB data set shown in Table 8, this work achieves 98.62% recognition accuracy, which is a comparable performance (1.03% and 0.03% less) to work by Jin [15] and Tang [22], and higher performance than the work by Zaklouta [17]. Considering the training time and recognition time (including the traffic sign detection and classification), this work improves 2 times and 8 times on the training speed and recognition speed, respectively, comparing with the work in Tang [22] even though our hardware platform is not as good as the hardware platform in Tang [22]. In Jin [15], the experiments are conducted on two Tesla C2075 GPUs, and a 6-core Intel(R) Core i7-3960X 3.3-GHz computer. Compared with Jin [15], this work greatly reduces the training time and recognition time, which is very important in safe driving assistance systems.

### 4.5. Performance Evaluated in Real Traffic Conditions

Considering real traffic conditions, where a 2000 frame image containing 200 Chinese traffic signs is used for testing, we present the average performance comparison in the Figure 7. As shown in Figure 7, the ROC curve denotes the false positive rate *versus* classification accuracy, and the work of Tang [22] is adopted as it exhibits higher performance than the other works mentioned above. We train the classification the hardware platform (Intel (R) Core(TM), Duo CPU, E7200@2.53GHz, 4 GB memory, and 32 bit operating system), and perform the recognition experiments on the embedded hardware platform (TI DM6467: DSP (1 GHz) +ARM (500 MHz), 256 M 32 bit DDR2 memory) to achieve a fair performance comparison. The comparisons of the computing time are shown in Table 10.

As shown in Figure 7, with the increase of the false positive rate, the classification accuracy becomes high. Though this work experienced a small performance loss (0.2% less) compared with the work by Tang [22]—as this work implements computing speed optimization which cause a loss of data precision—both works achieve high classification accuracy(>95%).

Besides, as shown in Table 10, this work achieves the recognition speed of 43 frames per second, which improves the recognition speed by 3.3 times compared with the work by Tang [22]. Meanwhile, the training time is reduced by 61%. In this work, in the real testing of traffic signs, the proposed method in this work correctly detects 195 traffic signs, which shows our work achieves high robustness in traffic sign recognition.

## 5. Conclusions

This paper proposes two optimizations for robust and fast traffic sign recognition. First, a rotation invariant binary pattern based feature in the affine space and Gaussian space is designed to achieve fast and robust traffic sign detection. It is an improvement of the ASIFT. The ASIFT algorithm exhibits the highest discriminative performance among the state-of-the-art features, but it is not practical because of its high computation complexity. Second, the techniques of artificial neutral network based feature dimension reduction and classification are proposed to reduce the recognition time. These techniques transform the large amounts of feature computations into a small amount of parameter computations. Testing the publicly available data set in real conditions shows that this work achieves fast processing speed and robust traffic sign recognition. However, when computing the rotation invariant binary pattern based feature, this work reduces the number of affine spaces for the traffic signs to reduce the computation cost, which creates limitations for some applications, with large viewpoint changes and a small recognition accuracy loss. Further work will enhance the rotation invariant binary based feature in the affine space so that higher robustness in the viewpoint can be achieved with a small computation cost.

## Figures and Tables

**Table 1.** Performance comparison for feature clustering.

**Method**	**Topologies**	**Clustering Accuracy**	**Computation Time**
K-means	/	100% (baseline)	0.6 s
ANN based K-means	16->16->1	99.59%	0.001 s
ANN based K-means	16->8->2	98.64%	0.0015 s
ANN based K-means	16->4->4	96.53%	0.001 s

**Table 2.** Performance comparison with 320 × 240 image size.

**Method**	**Topologies**	**Classification Accuracy**	**Computation Speed (fps)**
ANN	16->16->1	98.62%	500
ANN	16->8->2	97.44%	435
ANN	16->4->4	95.32%	500
SVM	Kernel based	98.64%	25
Random Forest	Decision tree based	97.54%	125
Full matching	Point to point	99.89%	0.1

**Table 3.** Performance comparison when changing the image size.

**Method**	**Performance Metric**	**Image Size**			

		**320 × 240**	**640 × 480**	**1280 × 720**	**1920× 1080**
ANN	CA	98.62%	98.60%	98.59%	97.55%
	CS(fps)	500	150	58	16

SVM	CA	98.64%	98.63%	98.60%	98.56%
	CS(fps)	25	7.3	2.6	0.52

Random Forest	CA	97.54%	97.52%	97.44%	97.41%
	CS(fps)	125	33	9.1	2.3

Full matching	CA	99.89%	99.86%	99.83%	99.81%
	CS(fps)	0.1	0.045	0.013	0.004

**Table 4.** Processing times.

**Image Subset**	**ASIFT**	**ASURF**	**This Work**	**Speed up over ASIFT**	**Speed up over ASURF**
Speed limit signs	810 ms	420 ms	31 ms	25.1×	12.5×
Unique signs	1300 ms	610 ms	54 ms	23.1×	10.3×
Danger Signs	1010 ms	530 ms	44 ms	22.0×	11.0×
Mandatory signs	580 ms	230 ms	12 ms	47.3×	18.2×
Derestriction signs	940 ms	490 ms	37 ms	24.4×	12.2×
Other prohibitory signs	856 ms	460 ms	38 ms	21.5×	11.1×

**Table 5.** Correctly matched points comparison.

**Image Subset**	**ASIFT**	**ASURF**	**This Work**
Speed limit signs	23	15	19
Unique signs	39	21	36
Danger Signs	31	24	29
Mandatory signs	31	24	29
Derestriction signs	89	70	84
Other prohibitory signs	32	26	31

**Table 6.** Comparison with other works for the GTSRB data set.

**Feature Classification**	**Dimension**	**Recognition Accuracy**	**Training Time**	**Recognition Speed**
HOG+ANN	36	96.77%	1740 s	50 fps
LBP+ANN	59	96.59%	1260 s	100 fps
SIFT+ANN	128	97.74%	1980 s	1 fps
SURF+ANN	64	97.46%	1680 s	2 fps
Proposed feature +ANN	64	98.62%	600 s	200 fps

**Table 7.** Comparison with other works for the STS data set.

**Feature Classification**	**Dimension**	**Recognition Accuracy**	**Training Time**	**Recognition Time**
HOG+ANN	36	95.41%	2349 s	12 fps
LBP+ANN	59	95.62%	1944 s	21 fps
SIFT+ANN	128	97.10%	2911 s	0.2 fps
SURF+ANN	64	97.52%	2014 s	0.5 fps
Proposed feature +ANN	64	98.33%	923 s	52 fps

**Table 8.** Comparison with other works for the GTSRB data set.

**Method**	**Hardware**	**Recognition Accuracy**	**Training Time**	**Recognition Time**
Jin [15]	GPU C2075&6-CORE i7-3960X@3.3 Ghz	99.65%	>7 h	>1 s
Zaklouta [17]	/	96.14%	/	<0.02 s
Tang [22]	E8400@3.0 Ghz	98.65%	3600 s	0.04 s
This work	E7200@2.53 Ghz	98.62%	1800 s	0.005 s

**Table 9.** Comparison with other works for the STS data set.

**Sign Type**	**Larsson [10]**		**This work**	

	**Recall**	**#FP**	**Recall**	**#FP**
Pedestrian crossing	98.0%	0	99.0%	0
Designated lane right	95.8%	0	96.1%	0
No standing or parking	100.0%	0	100.0%	0
50 kph	91.7%	2	93.2%	2
30 kph	95.8%	1	97.4%	1
Priority road	95.7%	0	96.78%	0
Give way	94.7%	0	95.9%	0

**Table 10.** Comparison in real conditions.

**Method**	**Recognition Hardware**	**Training Time**	**Recognition Time**
Tang [22]	TI DM6467	5400 s	10 fps
This work	TI DM6467	2100 s	43 fps
